# The genus *Anaphothrips* with one new species from China (Thysanoptera, Thripidae)

**DOI:** 10.3897/zookeys.668.12376

**Published:** 2017-04-12

**Authors:** Yanze Cui, Jinghui Xi, Jun Wang

**Affiliations:** 1 College of Plant Science, Jilin University, Changchun 130062, China

**Keywords:** *Anaphothrips*, China, new species, thrips

## Abstract

A key to six species of *Anaphothrips* known from China is provided, together with distribution information. *Anaphothrips dentatus* **sp. n.** is described and illustrated from Sanjiang Plain in northeastern China, based on one male and five apterous females. This species is unusual in having the posterior margin of tergite VIII with a craspedum of small teeth rather than long microtrichia.

## Introduction

Currently, 81 species are described in the genus *Anaphothrips* (ThripsWiki 2017), most being associated with species of Poaceae. A key to distinguish the genus from similar genera in China was provided by Mirab-balou et al. (2012), and five species have been recorded in this genus from China (Mirab-balou et al. 2012). The diagnosis of the genus includes the following character states: antennae 8- or 9-segmented, sense cone on segment IV forked, on segment III forked or simple; pronotum without long setae; metafurcal spinula absent; all tarsi 2-segmented; abdominal tergite VIII posterior margin with or without comb, some species with craspedum; sternites without discal setae; male abdominal sternites usually with pore plate (Mound and Masami 2009).

**Figure 1.**
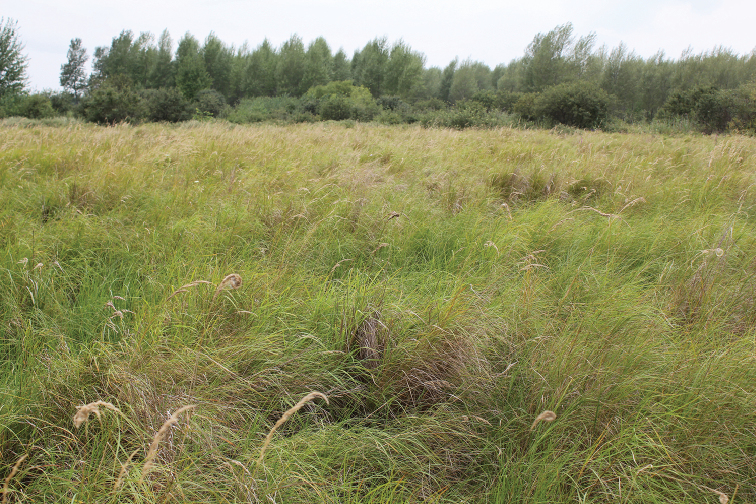
The habitats of *Anaphothrips dentatus* sp. n. (Photo taken on 9.viii.2012 by Jun Wang).

Sanjiang Plain (45°01.08'–48°27.93'N, 130°13.17'–135°05.43'E) is located in Northeast China, with a total area of approximately 108.9 thousand square kilometers. It is the largest area of freshwater marsh wetland (Fig. 1) in China, but from which no species of thrips has previously been reported. The thrips diversity of this region was investigated from 2012 to 2014, and two species from the genus *Anaphothrips* were discovered, *A. obscurus* (Müller) and the new species described in this paper.

## Materials and methods

The slide preparation method followed Zhang et al. (2006). Descriptions and measurements were conducted using a Nikon Eclipse 80i microscope; photographs were taken using an ISH500 camera with ISCapture software and were processed with the software of Adobe Photoshop CS6. All measurements described in this paper are in micrometers. One paratype of the new species is deposited in the Insect Collection, South China Agricultural University (**SCAU**); other specimens examined are deposited in the Insect Collection of Jilin University (**JLU**), Changchun City, Jilin Province, China.

## Taxonomy

### Anaphothrips beijingensis

Taxon classificationAnimaliaThysanopteraThripidae

Mirab-balou, Chen & Tong

Anaphothrips beijingensis Mirab-balou, Chen & Tong, 2012: 719.

#### Distribution.

China (Beijing).

### Anaphothrips dentatus sp. n.

Taxon classificationAnimaliaThysanopteraThripidae

http://zoobank.org/947ABA6E-BA6B-4B94-96B4-E5F8C509E0CD

Figs 2–8 , 9–14

#### Specimens examined.

Holotype: Female (apterous), China, Heilongjiang Province, Sanjiang Plain (47°35.08'N, 133°31.42'E), 18.vii.2013, from grasses (Jun Wang). Paratypes: 1 male and 1 female same data as holotype; 3 females, same locality and habitat as holotype, 2.vii.2014, from grasses (Jun Wang).

#### Diagnosis.

Both sexes apterous. Body brown, but head and thorax paler, legs yellow, antennal segments I, III–V yellow, segments II, VI–IX brown. Head wider than long slightly, projecting in front of eyes; ocelli reduced. Antennae 9-segmented, segments III–IV with sense cone forked. Pronotum almost smooth; metanotum median setae far apart and arising on posterior third of sclerite. Abdominal tergite VIII posterior margin with craspedum formed of small teeth. Male tergite IX with two pairs of stout median thorn-like setae near posterior margin; sternites III–VII with C-shaped pore plate slightly wider than distance between posteromarginal seta S1.

#### Description.

**Figures 2–8.**
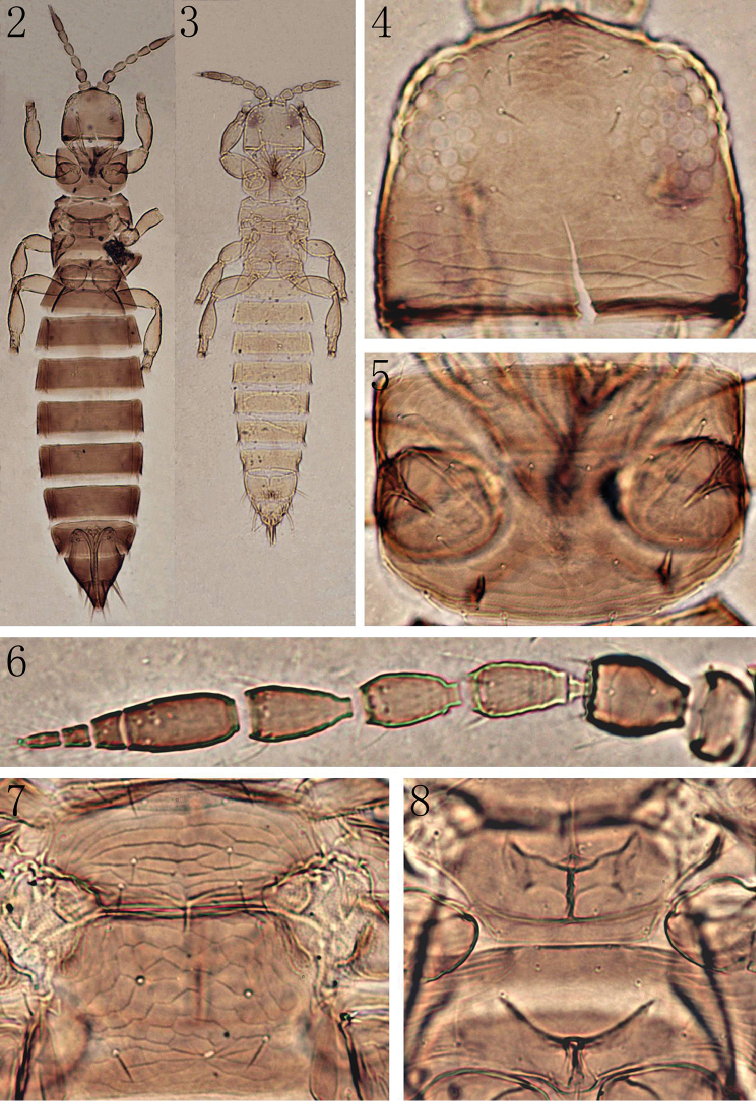
*Anaphothrips dentatus* sp. n. (**2, 4–8** female **3** male). **2** Female **3** Male **4** Head. **5** Pronotum. **6** Antenna **7** Meso- and metanotum **8** Meso- and metasternum.

**Apterous female** (Fig. 2). Body uniformly brown, head and thorax paler; legs yellow; antennal segments I, III–V yellow, segments II, VI–IX brown.

Head (Fig. 4) 0.9 times as long as wide, projecting in front of eyes, dorsal surface sculptured with irregular transverse reticulations, but almost smooth between eyes; ocelli reduced, three pairs of ocellar setae present, seta III longer; three pairs of postocular setae present; antennae 9-segmented (Fig. 6), segment III with pedicel, segments III–IV with small forked sense cone and rows of microtrichia, a complete transverse suture present between segments VI and VII.

**Figures 9–14.**
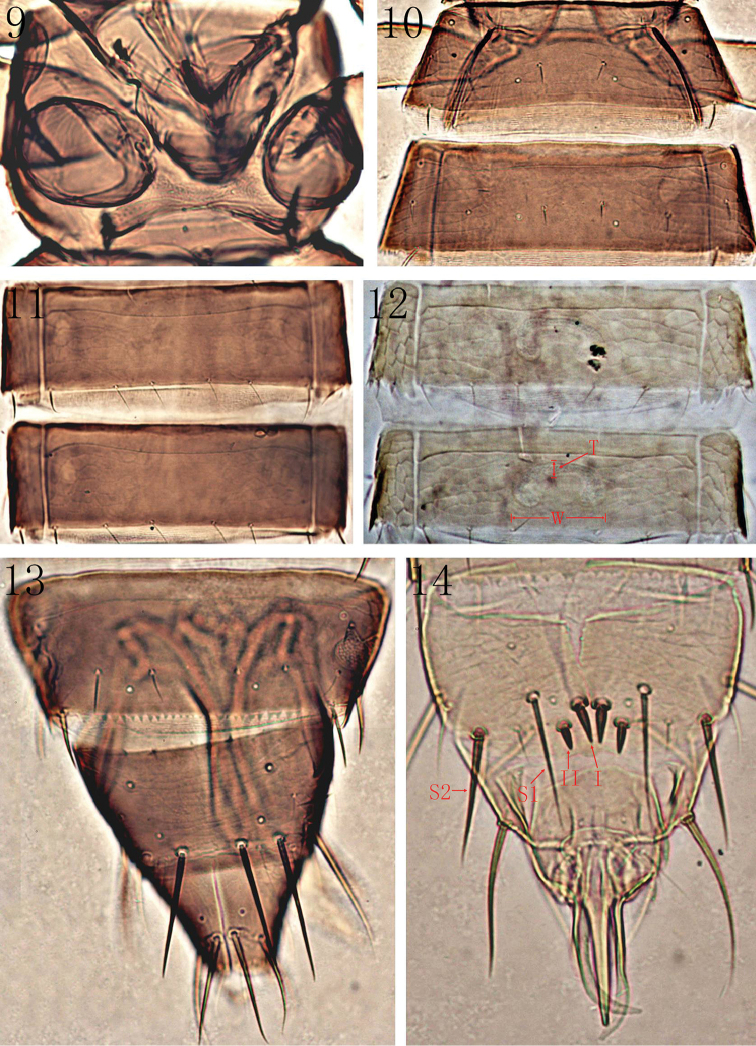
*Anaphothrips dentatus* sp. n. (**9–11, 13** female; **12, 14** male). **9** Ventral view of prothorax **10** Tergites II–III **11** Sternites IV–V **12** Sternites IV–V. T: thickness; W: width **13** Tergites VIII–X **14** Tergites IX–X.

Pronotum almost smooth (Fig. 5), without long setae; prosternal ferna undivided (Fig. 9). Mesonotum sculptured with transverse reticulations (Fig. 7), a pair of campaniform sensilla close to anterior margin, a pair of median setae and a pair of anterior external setae present, a pair of setae arising close to posterior margin. Metanotum sculptured with polygonal reticulations (Fig. 7), paired anteromarginal setae and paired campaniform sensilla present, paired median setae far apart and arising on posterior third of sclerite. Mesofurcal spinula present, metafurca without spinula (Fig. 8).

Abdominal tergites II–VIII with sculpture laterally (Fig. 10), one pair of median setae, two pairs of campaniform sensilla, two pairs of lateral setae and a pair of lateral marginal setae present, a pair of posteroangular setae arising at posterior margin far from the posterior angles; tergite VIII with spiracles occupying less than half of lateral margins, posterior margin with craspedum formed of small teeth; tergite IX with a pair of mid–dorsal setae and two pairs of campaniform sensilla, SI and S2 close to posterior margin longer than tergite X; tergite X divided longitudinally with a pair of campaniform sensilla and two pairs of long setae close to posterior margin (Fig. 13). Sternites II–VII reticulated laterally and without discal setae, sternite II with two pairs of posteromarginal setae, III–VII with three pairs (Fig. 11); sternite VIII with three pairs of setae laterally. Pleurotergites III–VII with posteromarginal setae, posterior margin with lobes (Fig. 11).

**Measurements** (holotype). Body length 1258. Head length 125, width 146; antennae length 238; antennal segments I–IX length(width): 20(28), 32(25), 37(17), 30(17), 31(18), 36(19), 8(11), 8(7), 12(4); antennal terminal setae 17; eyes length 66; diameter of ocelli 6, distance of posterior ocelli 37; ocellar setae I–III: 7, 7, 13; postocular setae I–III: 9, 13, 6. Pronotum median length 130, width 171; setae length 5–14. Mesonotum anterior median setae 8. Metanotum anteromarginal setae 12, median setae 14. Abdominal pelta median setae 11; tergite IX S1 65, S2 80; tergite X median setae 62; sternites II–VII posteromarginal setae 6–33.

**Apterous male** (Fig. 3). Similar to female but smaller and paler; tergite IX with two pairs of stout median thorn-like setae near posterior margin, setae I 1.7 times as long as II (Fig. 14); sternites III–VII with C-shaped pore plate slightly wider than distance between posteromarginal setae S1 (Fig. 12).

**Measurements** (paratype male). Body length 1003. Head length 120, width 141; antennae length 202; antennal segments I–IX length(width): 17(25), 27(22), 34(15), 26(15), 27(15), 30(15), 7(10), 6(7), 11(4); antennal terminal setae 14; eyes length 59; diameter of ocelli 5, distance of posterior ocelli 36; ocelli setae I–III: 9, 7, 11; postocular setae I–III: 7, 9, 8. Pronotum median length 106; width 152; setae length 3–11. Mesonotum anterior median setae 8. Metanotum anteromarginal setae 9, median setae 12. Abdominal tergite I median setae 12; tergite IX stout median thorn–like setae I 19, II 11, setae S1 53, S2 56; tergite X median setae 63; sternites II–VII posteromarginal setae 7–28; sternites III–VII pore plate thickness (T) 5–9, width (W) 48, 45, 47, 48, 36.

#### Distribution.

China (Heilongjiang Province).

#### Etymology.

The Latin word *dentatus* derived from tooth-shaped craspedum on abdominal tergite VIII posterior margin.

#### Remarks.

The new species belongs to a small group of *Anaphothrips* in which abdominal tergite VIII posterior margin has a craspedum that is tooth-shaped not ciliate. It is similar in appearance to the description by Pitkin (1978) of the Australian species *A. moundi*, but can be distinguished from the latter by the following features: antennal segment II brown, sensorium on III forked; abdominal tergite VIII with craspedum of teeth longer; male abdominal sternites pore plate only slightly wider than the distance of posteromarginal setae S1. The morphological characteristics of *A. moundi* are provided by Mound and Masumoto (2009). The new species is also similar to *A. obscurus* in appearance, but can be distinguished by the following characters: both sexes apterous; ocelli reduced; head wider than long; abdominal tergite VIII with tooth-shaped craspedum; male sternites III–VII with C-shaped pore plates only slightly wider than the distance of posteromarginal setae S1.

### Anaphothrips floralis

Taxon classificationAnimaliaThysanopteraThripidae

Karny

Figs 15–16

Anaphothrips floralis Karny, 1922: 109; Zhang and Tong 1992: 73.

#### Description.

**Figures 15–24.**
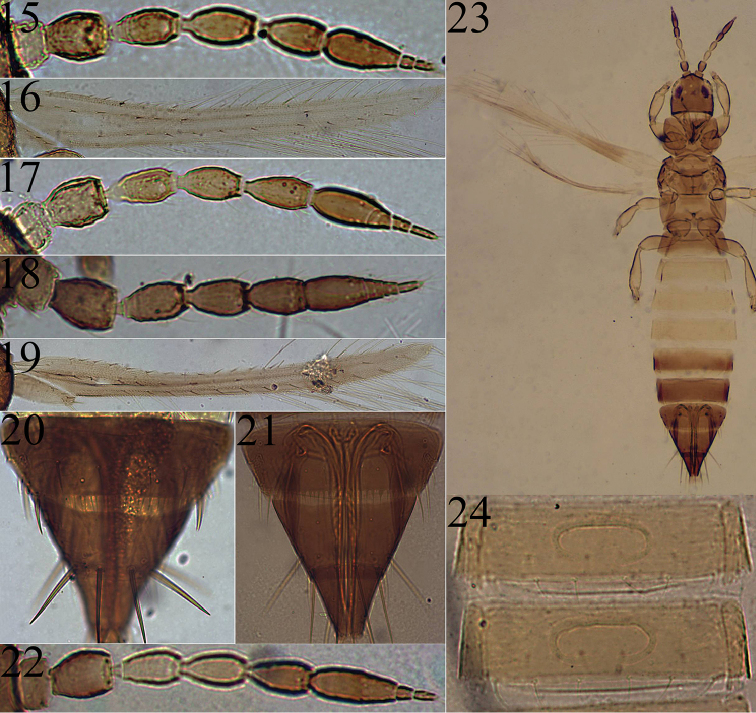
*Anaphothrips* of China. *A. floralis* (**15–16)**. **15** Antennae **16** Fore wing **17** *A. obscurus* antennae. *A. populi* (**18–20**) **18** Antennae **19** Fore wing **20** Abdominal tergites VIII–X. *A. sudanensis* (**21–23** female; **24** male) **21** Abdominal tergites VIII–X **22** Antennae **23** Body **24** Sternites IV–V.

**Macropterous female.** Body and legs yellow; antennal segment I yellow, segments II–IV and base of V light brown but segment II darker, segments V–VIII brown. Head with ocellar setae III arising at outer tangent between fore and hind ocelli; antennae 8-segmented (Fig. 15), segments III–IV with sensorium forked. Fore wing (Fig. 16) upper vein with eight basal setae and four distal setae, lower vein with 6–11 setae. Metanotum reticulate in mid line, campaniform sensilla absent. Abdominal tergite VIII posterior margin with complete comb.

#### Distribution.

China (Guangdong); Vietnam.

#### Specimens examined.

1 female (macropterous), 1 male (macropterous), China, Guangdong Province, suburb of Guangzhou, 4.xi.1976, form *Allium tuberosum* (Weiqiu Zhang).

### Anaphothrips obscurus

Taxon classificationAnimaliaThysanopteraThripidae

(Müller)

Fig. 17

Thrips obscura Müller, 1776: 96.

#### Description.

**Macropterous female**. Body and legs yellow; antennal segment I yellow, segments II-IV light brown but segment II darker, segments V-IX brown but segment V paler slightly; fore wing weakly shaded. Head wider than long, dorsal surface sculptured with irregular transverse reticulations behind eyes; ocellar setae I present, ocellar setae III out of ocellar triangle just anterior to hind ocelli. Antennae 9-segmented (Fig. 17), segments III–IV with sense cone forked. Fore wing upper vein with 7–8 basal setae and 2–3 distal setae, lower vein with 8–9 setae. Abdominal tergites with microtrichia laterally, tergite VIII posterior margin with comb of microtrichia.

**Micropterous male.** Similar to macropterous female, but wing shorter than thorax width (Mound and Masumoto 2009).

#### Distribution.

Widespread around the world.

#### Specimens examined.

3 female (macropterous), 5 female (micropterous), China, Heilongjiang Province, Sanjiang Plain, 2.vii.2014, from grasses (Jun Wang). 1 female (micropterous) China, Heilongjiang Province, Sanjiang Plain, 9.viii.2012, from grasses (Jun Wang). 1 female (micropterous), China, Ningxia, Pingluo, 24.vii.1987, from *Polygonum* (Caixia Yang).

### Anaphothrips populi

Taxon classificationAnimaliaThysanopteraThripidae

Zhang & Tong

Figs 18–20

Anaphothrips populi Zhang & Tong, 1992: 71.

#### Description.

**Macropterous female.** Body brown; antennae brown, segment I paler; all leg brown, tarsi paler; fore wing and clavus pale. Head about 0.7 times as long as wide. Ocellar setae 3 pairs, setae III arising at anterior margin of ocellar triangle almost as long as I and II. Antennae 9-segmented (Fig. 18), segments III and IV with forked sense cone. Fore wing (Fig. 19) upper vein with 8–9 basal setae and 3–4 distal setae, lower vein with 11–13 setae; clavus with 5 short setae. Abdominal tergites III–VII with irregular transverse sculpture laterally; tergite VIII (Fig. 20) posterior margin with comb of long microtrichia.

#### Remarks.

This species is similar to *A. incertus* in appearance but can be distinguished from the latter by the following features: antennal segments III–IV brown; segment VI with incomplete suture in distal third; fore wing upper vein with 8–9 basal setae and 3–4 distal setae, lower vein with 11–13 setae.

#### Distribution.

China (Henan, Gansu).

#### Specimens examined.

Paratype: 1 female (macropterous), China, Henan Province, Baiquan, Baoding Mountain, 21.vi.1979, from *Populus* (Shengfu Shi).

### Anaphothrips sudanensis

Taxon classificationAnimaliaThysanopteraThripidae

Trybom

Figs 21–24

Anaphothrips sudanensis Trybom, 1911: 1; Zhang and Tong 1992: 73.

#### Description.

**Macropterous female.** Body bicolored (Fig. 23), generally brown but abdominal segments III–V yellow; antennal segments I–II and V–VIII brown, segments III–IV yellow; legs yellow; fore wing pale but with dark cross band close to base. Head wider than long slightly. Antennae 8-segmented (Fig. 22), segments III–IV with forked sense cone. Pronotum weakly sculptured. Fore wing upper vein with six basal setae and five distal setae, lower vein with six setae; Abdominal tergite VIII (Fig. 21) posterior margin with comb of long microtrichia.

**Macropterous male.** Similar to female, but stergites III–VIII with large C-shaped pore plate (Fig. 24).

#### Distribution.

China (Hubei, Hunan, Jiangsu, Zhejiang, Fujian, Taiwan, Guangdong, Hainan, Guangxi, Sichuan, Guizhou, Yunnan); worldwide in tropical and sub-tropical countries.

#### Specimens examined.

1 female (macropterous), China, Guangdong Province, Guangning, Baoding mountain, 16.vii.2014, from *Pelargonium hortorum* (Zhaohong Wang). 1 male (macropterous), China, Guangdong Province, Guangning, Baoding Mountain, 16.vii.2014, from *Phyllanthus urinaria* (Zhaohong Wang).

### Key to Chinese species of *Anaphothrips*

(* not examined)

**Table:** 

1	Antennae clearly 8-segmented (Fig. 15)	**2**
–	Antennae 9-segmented (Fig. 17), segment VI with complete oblique or transverse suture	**4**
2	Female body distinctly bicolored (Fig. 23), mainly dark brown, antennal (Fig. 22) segments III–IV and abdominal segments III–V (or VI) yellow, male color various	***A. sudanensis***
–	Female body brown or yellow, not distinctly bicoloured	**3**
3	Head with ocellar setae III arising outside of ocellar triangle, anterior to hind ocelli; fore wing upper vein with 8–11 setae, lower vein with 7–9 setae; metanotum reticulate, campaniform sensilla present	***A. beijingensis*** *
–	Head with ocellar setae III arising at outer tangent between fore and hind ocelli; fore wing upper vein with about 12 setae, lower vein with 6–11 setae; metanotum reticulate in mid line, campaniform sensilla absent	***A. floralis***
4	Tergite VIII (Fig. 13) posterior margin with tooth-shaped craspedum; male sternal pore plates (Fig. 12) only slightly wider than distance between posteromarginal setae S1	***A. dentatus* sp. n.**
–	Tergite VIII (Fig. 21) posterior margin with comb of long microtrichia	**5**
5	Body yellow; antennal (Fig. 17) segment I yellow, segments II–IV light brown, segments V–VIII dark brown; microptera or else fore wing upper vein with 7–8 basal setae and 2–3 distal setae, lower vein with 8–9 setae	***A. obscurus***
–	Body brown; antennae (Fig. 18) brown, segment I paler; fore wing (Fig. 19) upper vein with 8–9 basal setae and 3–4 distal setae, lower vein with 11–13 setae	***A. populi***

## Supplementary Material

XML Treatment for Anaphothrips beijingensis

XML Treatment for Anaphothrips dentatus

XML Treatment for Anaphothrips floralis

XML Treatment for Anaphothrips obscurus

XML Treatment for Anaphothrips populi

XML Treatment for Anaphothrips sudanensis
